# Global emergence of Langya virus: A serious public health concern

**DOI:** 10.7189/jogh-13-03034

**Published:** 2023-07-07

**Authors:** Zahra Z Piracha, Umar Saeed, Rawal AI Ahmed, Fatima NA Khan, Muhammad I Nasir

**Affiliations:** 1 International Center of Medical Sciences Research (ICMSR), Islamabad, Pakistan; 2 Clinical and Biomedical Research Center, Foundation University School of Health Sciences (FUSH), Foundation University Islamabad, Pakistan; 3 Regional Disease Surveillance and Response Unit Sukkur, Sindh, Pakistan; 4 Baqai Medical University Karachi, Karachi, Pakistan; 5 Fazaia Ruth Pfau Medical College – FRPMC PAF Base Faisal Karachi

## EMERGENCE OF LANGYA VIRUS DURING COVID-19 PANDEMIC IS A THREAT TO GLOBAL COMMUNITIES

The newly-emerged Langya virus (LayV), classified as a henipavirus in the *Paramyxoviridae* family and closely related to the Mojianghenipavirus, has raised concerns among public health authorities worldwide. The coronavirus 2019 (COVID-19) pandemic, which started in China in 2019, is still ongoing, despite joint global efforts [1]. The ensuing global crisis has severely impacted the world’s economy, affecting supply chains for medicines, food, and materials across low-, middle-, and high-income countries, most of which still have to recover. The simultaneous re-emergence of a life-threatening LayV from China raises serious questions and necessitates in-depth clinical investigations to prevent future pandemics. Timely diagnosis of LayV infections and the use of established prevention methods can significantly reduce the global burden of diseases [2].

## HUMAN-TO-HUMAN TRANSMISSION OF LANGYA VIRUS NEEDS EXTENSIVE RESEARCH

Most disease outbreaks during the past two decades have been of zoonotic origin [3]. Although as many as 10 000 different species of viruses could potentially affect human beings, most commonly circulate in wild animals [4]. Rapid climate changes and increased human activity has increased the risk of viral exchange across different species of animals, which occasionally results in the transmission of zoonotic diseases to human beings [5]. 

LayV has emerged as a threat following the Hendra and Nipah viruses, taking its name after a town called Langya in China. From its emergence in 2018, 35 cases of LayV infection have been reported, all in eastern China. To date, no deaths have been reported. Despite its low global prevalence, this novel virus could lead to a global public health crisis, especially due to the potential high mortality from earlier species of the henipavirus genus, which is why it cannot be underestimated [7,8]. Currently, the LayV spreads from animals to human beings. Although contact tracing and investigation discovered no person-to-person transmission cases, this cannot be excluded due to the small sample size. LayV symptoms include fever, cough, tiredness, gastrointestinal disturbance, loss of appetite, muscle pain, nausea, and vomiting. Most cases had leukopenia, pointing to their compromised immunity against infections. The second most common sign was a decreased number of platelets. Some of the infected individuals also exhibited hepatic symptoms, hinting at the LayV’s possible effect on the liver [9]. The virus has recently been linked to the transfer of infections from animals to people in Eastern China. It was also discovered during COVID-19 and Monkeypox investigation and procedures [10]. However, human-to-human transmission of LayV has not yet been recognised.

Langya virus is an enveloped virus containing single-stranded ribonucleic acid (RNA) with 18 402 RNA nucleotides. Its genomic structure consists of six structural proteins – nucleocapsid, phosphoprotein, matrix protein, surface glycoprotein, fusion protein, and large viral RNA-dependent RNA polymerase. These six structural proteins determine its closest genetic linkage with other henipaviruses like Mojiang henipavirus [8]. The detailed genomic sequences of LayV can be found in the GenBank (accession numbers OM101125- OM101130 and OM069567-OM069646).

As previously mentioned, the virus primarily transmits between animals, most likely among shrews. The LayV RNA was isolated from more than 25% of almost 260 shrews studied, leading researchers to conclude that the species may possibly serve as a direct or indirect vector for human transmission. The LayV RNA has also been isolated from other animals, including dogs and goats [7]. Among 35 identified human infections so far, none seems to be the result of human-to-human transmission. However, the high prevalence of LayV among shrews is alarming, necessitating large-scale studies to determine possible transmission routes of LayV. 

## GLOBAL PERSPECTIVES ON LANGYA VIRAL PREVENTION AND MANAGEMENT

Farmers and manufacturing workers are the populations most vulnerable to LayV [7,11], as research has shown that proximity to animals and working in agricultural settings are linked to LayV virus infection. To stop LayV from spreading, monitoring must be strengthened to enable early discovery [12]. There is no particular therapy for LayV, akin to henipaviruses and other viruses with similar clinical presentation; few antiviral drugs have been tested by experts in animal experiments, and there is no specific vaccine. However, ribavirin could be an effective remedy. It is frequently prescribed by healthcare providers for treating viral infections and is beneficial against RNA viruses and those that cause respiratory symptoms. Research has shown that ribavirin is also effective against both Hendra and Nipah viruses. These two viruses can also be treated with chloroquine, which is a tested medication for malaria. Based on available data, these two treatments may aid in the management of the Langya virus [11,12]. Viral infections are mounting over time [13-23], so prevention at all levels against viruses – including the novel LayV – remains the optimal strategy. As with any other zoonotic agent, holistic knowledge and adequate preparedness will be our best defence. Future studies should provide evidence regarding possible transmission routes, pathological changes, vulnerable groups, potential reservoirs, susceptible hosts, and rapid viral diagnostic advances via artificial intelligence tools, to ensure the effective containment of this new virus. We call for international and national health strategic organisations to prevent zoonotic diseases spread and design timely prevention strategies against Langya viral transmission.
